# Reliability of ultrasound to measure morphology of the toe flexor muscles

**DOI:** 10.1186/1757-1146-6-12

**Published:** 2013-04-04

**Authors:** Karen J Mickle, Christopher J Nester, Gillian Crofts, Julie R Steele

**Affiliations:** 1 Centre for Health Sciences Research, University of Salford, Salford, UK; 2 Biomechanics Research Laboratory, University of Wollongong, Wollongong, Australia

**Keywords:** Anatomy, Foot structure, Muscle atrophy, Ultrasonography

## Abstract

**Background:**

Measuring the strength of individual foot muscles is very challenging; however, measuring muscle morphology has been shown to be associated with strength. A reliable method of assessing foot muscle atrophy and hypertrophy would therefore be beneficial to researchers and clinicians. Thus, the aim of this study was to evaluate the test-retest intra-observer reliability of ultrasound to measure the morphology of the primary toe flexor muscles.

**Method:**

The abductor hallucis, flexor hallucis brevis, flexor digitorum brevis, quadratus plantae and abductor digiti minimi muscles in the foot, and the flexor digitorum longus and flexor hallucis longus muscles in the shank were assessed in five males and five females (mean age = 32.1 ± 10.1 years). Muscles were imaged using a GE Venue 40 ultrasound (6-9 or 7.6-10.7 MHz transducer) in a random order, and on two occasions 1-6 days apart. Muscle thickness and cross-sectional area were measured using Image J software with the assessor blinded to muscle and day of scan. Intraclass correlation coefficients (ICC) and limits of agreement were calculated to assess day-to-day repeatability of the measurements.

**Results:**

The method was found to have good reliability (ICC = 0.89-0.99) with limits of agreement between 8-28% of the relative muscle size.

**Conclusion:**

The protocol described in this paper showed that ultrasound is a reliable method to measure morphology of the toe flexor muscles. The portability and advantages of ultrasound make it a useful tool for clinical and research settings.

## Background

Strength of the toes and somatosensory information from the plantar surface of the foot and ankle are important factors for safe ambulation and standing balance [1]. In particular, the intrinsic foot muscles provide an essential role in stabilising the foot and arch. Therefore, maintaining adequate intrinsic foot muscle strength is imperative for efficient performance of activities of daily living. From a sporting perspective, increased strength of the toe flexor muscles has been found to contribute to enhancing athletic performance [2]. Conversely, atrophy of the plantar foot muscles, and the associated development of an imbalance between the flexor and extensor muscles, is believed to be a primary cause of toe deformities such as claw and hammer toes, and prominent metatarsal heads [3]. Loss of normal toe function is thought to play an important role in changes in gait because the toes have the longest lever arm around the ankle of any foot structure [4]. Indeed, toe deformity has been found to double the risk of falling in older people [5]. Normal toe dorsiflexion is also critical in developing tensile forces in many plantar structures that support the multiple and highly mobile joints of the foot [6].

Despite their importance, measuring the functional performance of individual foot muscles is very challenging. Muscle morphology, however, has been shown to be indicative of muscle performance, including strength [7,8]. Furthermore, ankle dorsiflexion and plantar flexion strength have been found to significantly correlate with volume and cross-sectional area (CSA) of the corresponding muscle groups [8]. For example, a strong relationship has been found between maximal voluntary contraction strength and muscle CSA of the ankle dorsiflexor muscles (r = 0.81) in sedentary older adults [9]. To date, however, few studies have reported in vivo muscle architecture of the intrinsic foot muscles. A recent review by Soysa et al. [10] provides a good review of the challenges of measuring intrinsic foot muscle strength.

Due to its high spatial resolution, magnetic resonance imaging (MRI) has been used successfully to detect atrophy of the intrinsic foot muscles [11-13]. However, MRI is time consuming, expensive and cannot be performed in many clinical or community settings. Despite the potential for important functional changes to be caused by foot muscle atrophy, no clinical methods for evaluating plantar muscle morphology have been established. As a result, little is known about the causes and progression of foot muscle atrophy, or whether it can be reversed through rehabilitation. A reliable method of assessing foot muscle atrophy and hypertrophy would therefore be beneficial to researchers and clinicians.

Real-time ultrasound (US) is a non-invasive, objective and inexpensive method of assessing muscle morphology and has been employed widely to quantify cross-sectional area and linear dimensions of larger lower limb muscles (e.g. quadriceps, triceps surae, anterior tibial muscle group) [14,15]. Few studies, however, have determined its ability to measure the small muscles of the foot and ankle, and repeatability has only been reported for the abductor hallucis muscle [16,17]. Therefore, this study aimed to describe a protocol to identify the primary toe flexor muscles and determine whether ultrasound is a reliable tool to measure the morphology of the toe flexor muscles. Understanding the muscle architecture of the foot has implications for rehabilitation, the analysis of normal foot function, biomechanical modelling, as well as the design of footwear, prostheses and orthotics.

## Methods

A convenience sample of five males and five females (mean age = 32.1 ± 10.1 years) was recruited from a university student and staff population. Participants were required to be over the age of 18 years and have no lower limb disorders. Written informed consent was obtained from each participant and their rights were protected throughout the study. Ethics approval was obtained from the University’s Research Ethics Panel (REP10/062).

The abductor hallucis, flexor hallucis brevis, flexor digitorum brevis, quadratus plantae and abductor digiti minimi muscles in the foot and the flexor digitorum longus and flexor hallucis longus muscles in the shank were the muscles of interest as they are the main contributors to toe flexion. Extensive pilot testing, with reference to anatomy textbooks, models and published cadaver, ultrasound and MRI studies [18-20] was conducted by the chief investigator [KJM] before the study, to determine the best scanning protocol. Muscles were imaged on two occasions, 1-6 days apart (mean = 3.5 ± 2.2 days). A portable Venue 40 musculoskeletal ultrasound system (GE Healthcare, United Kingdom) fitted with either a 6-9 (maximum depth 5 cm) or 7.6-10.7 MHz (maximum depth 3 cm) linear transducer, was used to image the toe flexor muscles of each participant. The scanning was conducted on each participant’s dominant stance limb (i.e. the leg chosen by each participant when they were asked to perform a single-leg balance task).

**Figure 1:**
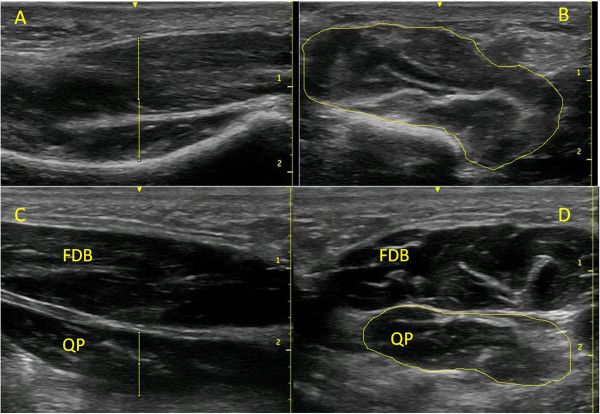
**Example images taken of the flexor hallucis brevis, flexor digitorum brevis and quadratus plantae muscles.** (**A**) Longitudinal view of the flexor hallucis brevis; (**B**) cross-sectional area of flexor hallucis brevis; (**C**) longitudinal view of the flexor digitorum brevis (FDB) and quadratus plantae (QP) muscles; and (**D**) cross-sectional area of the flexor digitorum brevis (FDB) and quadratus plantae (QP) muscles. Scale is in cm.

**Figure 2:**
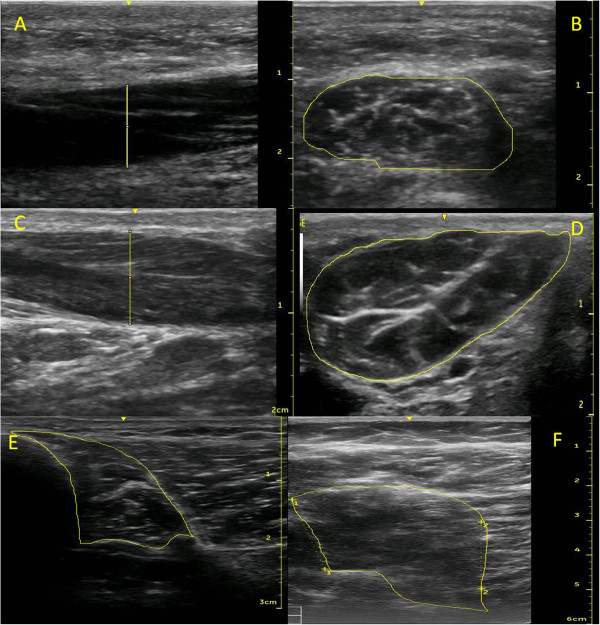
**Example images taken of the abductor digiti minimi, abductor hallucis, flexor digitorum longus and flexor hallucis longus muscles.** (**A**) Longitudinal view of abductor digiti minimi; (**B**) cross-sectional area of abductor digiti minimi; (**C**) longitudinal view of the abductor hallucis; and (**D**) cross-sectional area of abductor hallucis; (**E**) Cross-sectional area of the flexor digitorum longus; and (**F**) cross-sectional area of flexor hallucis longus. Scale is in cm.

For the flexor digitorum brevis, quadratus plantae, flexor hallucis brevis and abductor digiti minimi muscles, participants lay prone on a plinth with their feet hanging freely. To locate flexor hallucis brevis, the transducer was aligned longitudinally along the shaft of the 1st metatarsal and scanned in a proximal direction until the thickest portion of the muscle belly was located, distal to the base of the metatarsal. A still image was captured and used to measure the thickness of the muscle (Figure 1A). The transducer was then rotated 90° to obtain the cross-sectional image of the muscle (Figure 1B). To locate the major muscle belly of flexor digitorum brevis, a line joining the medial tubercle of the calcaneus to the 3rd toe was drawn on the plantar surface of the foot. The transducer was placed longitudinally on this line at the muscle’s insertion into the calcaneus and the muscle was scanned in a distal direction until the thickest portion of the muscle belly, before it divided into its four muscle fascicles, was found. A still image was captured and used to measure the thickness of the muscle (Figure 1C). The transducer was then rotated 90° to obtain the cross-sectional image of the muscle (Figure 1D). Quadatus plantae, lying deep to flexor digitorium brevis, was imaged by locating the talocalcaneonavicular joint in the sagittal plane and aligning the probe longitudinally along the muscle fibres. The thickest part of the muscle belly was found, often just proximal to the spring ligament, and a still image was captured and used to measure the thickness of the muscle (Figure 1C). The transducer was then rotated 90° to obtain the cross-sectional image of the muscle (Figure 1D). To locate abductor digiti minimi, the probe was placed at the muscle’s origin on the lateral calcaneal tuberosity, directed towards the tuberosity of the 5th metatarsal. The thickest part of the muscle was located, typically near the calcaneo-cuboid joint, before the appearance of the tendon, and a still image was captured and used to measure the thickness of the muscle (Figure 2C). The transducer was then rotated 90° to obtain the cross-sectional image of the muscle (Figure 2D).

To access the abductor hallucis, flexor digitorum longus and flexor hallucis longus, participants lay supine with their hip externally rotated and knee slightly flexed. To locate abductor hallucis, the probe was placed at the muscle’s origin on the medial calcaneal tuberosity directed towards the navicular tuberosity. The thickest part of the muscle was located, typically 1-2 cm proximal to the navicular tuberosity, and a still image was captured and used to measure the thickness of the muscle (Figure 2A). The transducer was then rotated 90° to obtain the cross-sectional image of the muscle (Figure 2B). A location for measuring the cross-sectional area of abductor hallucis was assessed in order to allow later comparisons with the results of Cameron et al. [16]. A scanning line was drawn at the anterior aspect of the medial malleolus, perpendicular to the long axis of the foot. The transducer was placed along this line and a still image was captured and used to measure the cross-sectional area of the muscle.

The long toe flexor muscles, flexor digitorum longus and flexor hallucis longus, were imaged at two locations with scans taken at 40 and 50% of the distance from the inferior margin of the medial malleous to the medial tibial condyle. After measuring tibia length, scanning lines were drawn at 40 and 50%, perpendicular to the long axis of the shank. The transducer was placed on the medio-posterior surface of tibia. Flexor digitorum longus was imaged at this location, the muscle sitting adjacent to the tibia, deep to soleus (Figure 2E). To image flexor hallucis longus, the transducer remained on the scanning line, but was moved a few centimetres posteriorly, deep to gastrocnemius and soleus (Figure 2F).

For all described above measurement sites, ultrasound coupling gel was applied over the skin and transducer. To optimise image quality, the transducer was positioned so that the ultrasound beams were aimed perpendicular to the muscle borders. Depth and gain were adjusted to obtain a satisfactory image. Participants were asked to actively contract selected muscles to help identification (e.g. “push down with your big toe against my finger”), and then the image was captured when muscles were in a relaxed state. The tester applied minimal pressure to the ultrasound probe in order to reduce deformation of the muscle and surrounding tissues. Three measurements were taken at each site, removing the probe between each trial. All the scans were taken by the chief investigator [KJM], who has 8 years of research experience in musculoskeletal ultrasound.

The images were stored and transferred to a computer for measurement. Muscle thickness (cm) and cross-sectional area (cm^2^) were measured using Image J software (National Institute for Health, Bethesda, MD, USA) with the assessor [KJM] blinded to muscle and day of scan. The use of this software to measure muscle thickness from ultrasound images has been shown to have excellent inter-rater reliability [15].

The mean of three measurements was calculated for each muscle and day. Intraclass correlation coefficients (ICC; 3,1) were then calculated using SPSS (Version 17, SPSS Inc., Chicago, IL) to assess day-to-day reliability of the measurements. Limits of agreement (LoA) were also calculated (mean difference ± 1.96 x standard deviation), as described by Bland and Altman [21], whereby lower values indicate better agreement between days.

## Results

The descriptive characteristics of the measured muscles and their corresponding ICC and LoA values are listed in Table 1. With the exception of flexor hallucis longus at 40% of tibial length, between-session reliability for measuring muscle size was deemed moderate (ICC > 0.8) or high (ICC > 0.9) [22]. Again, with the exception of flexor hallucis longus at 40% of tibial length, the limits of agreement for all measurements were between 8 and 28% of the relative muscle size.

**Table 1:** **Mean (± SD) muscle thickness (cm) and cross-sectional area (CSA; cm**^**2**^**) values taken on Day 1 and 2 and their respective Intraclass Correlation Coefficient (ICC) and absolute Limits of Agreement (LoA) values with their normalised values in parentheses**

	**Day 1**	**Day 2**	**ICC**	**LoA (%)**
ABH CSA	2.56 ± 0.89	2.46 ± 0.87	0.95	0.75 (30)
ABH thickness	1.24 ± 0.34	1.23 ± 0.38	0.98	0.22 (18)
ABH CSA_MM_	2.45 ± 0.94	2.43 ± 0.83	0.98	0.42 (17)
FDB CSA	2.10 ± 0.54	2.19 ± 0.54	0.99	0.18 (8)
FDB thickness	1.00 ± 0.19	1.02 ± 0.20	0.95	0.16 (16)
ABDM CSA	2.11 ± 0.64	2.10 ± 0.61	0.98	0.33 (16)
ABDM thickness	1.08 ± 0.32	1.05 ± 0.26	0.97	0.19 (18)
FHB CSA	2.45 ± 0.53	2.49 ± 0.59	0.89	0.69 (28)
FHB thickness	1.28 ± 0.31	1.21 ± 0.28	0.97	0.21 (17)
QP CSA	1.74 ± 0.60	1.75 ± 0.55	0.99	0.16 (9)
QP thickness	1.04 ± 0.27	1.01 ± 0.25	0.97	0.18 (18)
FDL CSA_50%_	1.73 ± 0.39	1.59 ± 0.42	0.98	0.23 (14)
FDL CSA_40%_	1.76 ± 0.42	1.68 ± 0.37	0.92	0.43 (25)
FHL CSA_50%_	3.83 ± 0.95	4.15 ± 0.94	0.98	0.48 (11)
FHL CSA_40%_	4.28 ± 0.62	4.34 ± 0.74	0.57	1.31 (30)

ABH = abductor hallucis; MM = taken at medial malleous; FDB = flexor digitorum brevis; ABDM = abductor digiti minimi; QP = quadratus plantae; FDL = flexor digitorum longus; FHL = flexor hallucis longus; 40% and 50% refer to measurement taken at 40 and 50% of the tibial length, respectively.

## Discussion

Characterising muscle morphology has in the past been difficult, expensive and time-consuming. The availability of relatively low-cost and portable ultrasound machines now makes this more feasible, especially when investigating or caring for populations in community or physical therapy settings. However, in order for a measurement technique to be deemed useful, the method must be proven to be reliable. This study has shown that ultrasound is a reliable method to measure morphology of the toe flexor muscles.

In the only other study to have reported on the reliability of ultrasound to measure foot muscles, Cameron et al. [16] reported good between-session reliability (ICC = 0.79-0.97) for measuring the thickness, width and cross-sectional area of abductor hallucis. They reported an average cross-sectional area of 2.69 ± 0.35 cm, which is slightly larger than at the same anatomical location in the present study (2.44 ± 0.86 cm). They reported the muscle thickness adjacent to the medial malleolus to be 1.15 ± 0.1 cm, which is very similar to the thickest portion of the muscle belly in the present study (1.23 ± 0.34 cm). Taking an average across both days, as expected, the cross-sectional area measured at the thickest portion of the abductor hallucis muscle was larger than when measured at the medial malleolus (2.51 cm vs 2.44 cm), although this was not statistically different. Therefore, given the higher ICC and lower limits of agreement with using the anatomical landmark (0.98 and 0.42 cm, respectively) compared with trying to locate the thickest portion (0.95 and 0.75 cm), the former method is preferred. Interestingly, the thickest part of the muscle belly occurred at the same location as the medial malleolus measurement in two of the participants. The abductor hallucis, flexor digitorium brevis and abductor digiti minimi are bulky muscles that can be easily recognised with ultrasound imaging. Although smaller in size, the quadratus plantae, located in the second muscular layer, and flexor hallucis brevis, in the third layer, could also be easily identified.

Measures of anterior tibial muscle thickness using transverse and longitudinal approaches have been found to be comparable [15]. Longitudinal scans provide several measurements along the length of the muscle belly offering an excellent opportunity to locate the thickest section. In contrast, transverse images only contain once slice of the muscle. For flexor hallucis brevis, measuring muscle belly thickness was more reliable than measuring cross-sectional area. The lateral borders of this muscle were often difficult to identify, possibly due to thinner and oblique orientated epimysium not producing an echogenic envelope around the entire muscle belly. The lateral borders of the flexor digitorum brevis were clear in all cases and this was reflected in the cross-sectional area measurement being highly reliable. In some cases, the quadratus plantae displayed poorer lateral resolution, although repeatability of this measurement was still very high. Cameron et al. [16] reported better reliability for measuring the thickness of the abductor hallucis muscle (ICC = 0.97) compared to the cross-sectional area (ICC = 0.79).

The long toe flexor muscles flexor digitorum longus and flexor hallucis longus originate in the lower leg, cross over the ankle and metatarsal joints and insert onto the distal phalanges of the lesser digits and hallux, respectively. Their function is to control the flexion of distal phalanges of lesser toes or hallux as well as plantar flexing the ankle. After extensive pilot testing, two sites were chosen for the reliability study, 40% and 50% of tibia length. The repeatability of the measurements was superior at the 50% location and therefore should be used in future protocols. However, it is possible that the inaccuracy of the 40% measurement site occurred due to the specifications of the transducer used. The cross-sectional area of deeper flexor hallucis longus was larger at 40%, however the transducer had a maximal depth of 6cm which placed the muscle towards the lower boundaries of the field of view. Therefore a lower frequency probe which allows for greater depth may improve visualisation and reliability of the measurement. Despite extensive pilot testing, the deep lumbricals and plantar interosseous muscles in the foot proved very difficult to consistently detect. These muscles have the smallest muscle volume of all the foot muscles [20], and poor lateral resolution of the muscles did not produce a echogenic border. Severinsen et al. [23] used ultrasound to measure the thickness of muscles in the first interstitium, but the first dorsal interosseus muscle, transverse adductor hallucis muscle and first lumbrical muscle were grouped together as segmentation of individual muscles was not feasible.

Although published data is not available for all muscles tested, the limits of agreement were within the ranges that we might expect for changes in muscle size due to ageing, disease or intervention. For example, the extensor digitorum brevis muscle of diabetic patients has been found to be 46% smaller in cross-sectional area and 29% thinner, and the thickness of the muscle group between the 1st and 2nd metatarsal bones was 26% smaller [23]. Similarly, Bus et al. [12] reported a 73% reduction in the cross-sectional area of the forefoot muscles in diabetic patients compared to controls using MRI. A 7.3% increase in the thickness of the anterior tibial muscle group was found between the kicking and non-kicking limbs of Gaelic footballers [15]. A limitation of ultrasonography is that the lower resolution is less capable of identifying fatty infiltration of muscles in comparison to MRI. Therefore, ultrasound may overestimate muscle size and underestimate muscle atrophy, although the cases in which fatty infiltration occurs, is still not clear. However, this is unlikely to have affected the present investigation or the reliability of the method. The cross-sectional areas for flexor digitorum brevis, quatratus plantar, abductor digiti minimi and abductor hallucis are very similar to those reported in a large MRI study of 160 participants [24], supporting the validity of our ultrasound method.

It is acknowledged that only measuring at one location of a muscle does not fully characterise the morphology of the entire muscle. However, studies have shown a strong correlation between single muscle measures and muscle volume [23]. Specific to the intrinsic foot muscles, the thickness and cross-sectional areas obtained by ultrasound for the extensor digitorum brevis muscle and the muscle group between the 1st and 2nd metatarsal bones were highly correlated to the volume of the muscle obtained from MRI (r = 0.71 – 0.77) [23]. The protocol used in the present study aimed to locate the thickest portion of the muscle belly as hypertrophy of the anatomical cross-sectional area typically occurs at the region of maximal muscle girth (normally the central region) with little or no changes towards the proximal and distal ends [25]. Finally, it is acknowledged that this reliability study was conducted on a relatively small sample of 10 healthy men and women between the ages of 23 and 50 years, and therefore should be interpreted within the context of the population.

## Conclusion

The abductor hallucis, flexor hallucis brevis, flexor digitorum brevis, quadratus plantae and abductor digiti minimi muscles in the foot and the flexor digitorum longus and flexor hallucis longus muscles in the shank can be imaged using ultrasound. The protocols described in this paper to locate the muscle bellies were found to be repeatable across days. Ultrasound is therefore deemed a reliable method to measure morphology of the toe flexor muscles.

## Competing interests

KJM, CJN, GC and JRS have no competing interests to declare.

## Authors’ contributions

KJM, CJN and GC designed the study. KJM collected and analysed the data. KJM conducted the statistical analysis. KJM drafted the manuscript with assistance from CJN, GC and JRS. All authors read and approved the final manuscript.
